# PPINGUIN: Peptide Profiling Guided Identification of Proteins improves quantitation of iTRAQ ratios

**DOI:** 10.1186/1471-2105-13-34

**Published:** 2012-02-16

**Authors:** Chris Bauer, Frank Kleinjung, Dorothea Rutishauser, Christian Panse, Alexandra Chadt, Tanja Dreja, Hadi Al-Hasani, Knut Reinert, Ralph Schlapbach, Johannes Schuchhardt

**Affiliations:** 1 MicroDiscovery GmbH, Marienburger Str. 1, 10405 Berlin, Germany; 2 Functional Genomics Center, UNI ETH Zurich, Winterthurerstrasse 190, CH-8057 Zurich, Switzerland; 3 German Institute of Human Nutrition, Department of Pharmacology, Arthur-Scheunert-Allee 114-116, 14558 Nuthetal, Germany; 4 German Diabetes-Center at the Heinrich-Heine-University Düsseldorf; 5 Free University of Berlin, Department Computer Science and Mathematics, Berlin, Germany

## Abstract

**Background:**

Recent development of novel technologies paved the way for quantitative proteomics. One of the most important among them is iTRAQ, employing isobaric tags for relative or absolute quantitation. Despite large progress in technology development, still many challenges remain for derivation and interpretation of quantitative results. One of these challenges is the consistent assignment of peptides to proteins.

**Results:**

We have developed Peptide Profiling Guided Identification of Proteins (PPINGUIN), a statistical analysis workflow for iTRAQ data addressing the problem of ambiguous peptide quantitations. Motivated by the assumption that peptides uniquely derived from the same protein are correlated, our method employs clustering as a very early step in data processing prior to protein inference. Our method increases experimental reproducibility and decreases variability of quantitations of peptides assigned to the same protein. Giving further support to our method, application to a type 2 diabetes dataset identifies a list of protein candidates that is in very good agreement with previously performed transcriptomics meta analysis. Making use of quantitative properties of signal patterns identified, PPINGUIN can reveal new isoform candidates.

**Conclusions:**

Regarding the increasing importance of quantitative proteomics we think that this method will be useful in practical applications like model fitting or functional enrichment analysis. We recommend to use this method if quantitation is a major objective of research.

## Background

Quantitative proteomics is becoming increasingly important and over the last years many efforts have been made to develop and improve methods allowing for protein quantification. Besides gel based approaches [1,2], mass spectral techniques encompassing labeling techniques such as iTRAQ [3], ICAT [4] and SILAC [5,6] as well as label free approaches are widely-used for quantitative proteomics. Especially iTRAQ (isobaric tags for relative and absolute quantitation) gained much popularity as it allows for multiplexing quantitation of up to 8 samples. This new flexibility has been used recently in several studies investigating various objectives [7-11].

Complementing these experimental technologies, a wide range of quantification algorithms can be found in the literature. The most common algorithms are included in software packages such as MASCOT, ProQUANT, i-TRACKER [12,13], Multi-Q [14] or virtual expert mass spectrometrist (VEMS) [15]. In 2008 Lacerda et al. [16] compared the two software packages MASCOT and Peaks (Bioinformatics Solutions Inc., Waterloo, ON, Canada) [17] using a six-protein mixture as well as a complex protein sample. They revealed significant differences in the two packages as for a complex protein mixture only 26% of the proteins agreed within 20% error of quantitation ratios. The highest fold changes measured with iTRAQ differ widely among laboratories but rarely seem to exceed ten-fold, which was reported by Casado-Vela et al. [18] in a technical survey examining more than 200 articles.

**Figure 1:**
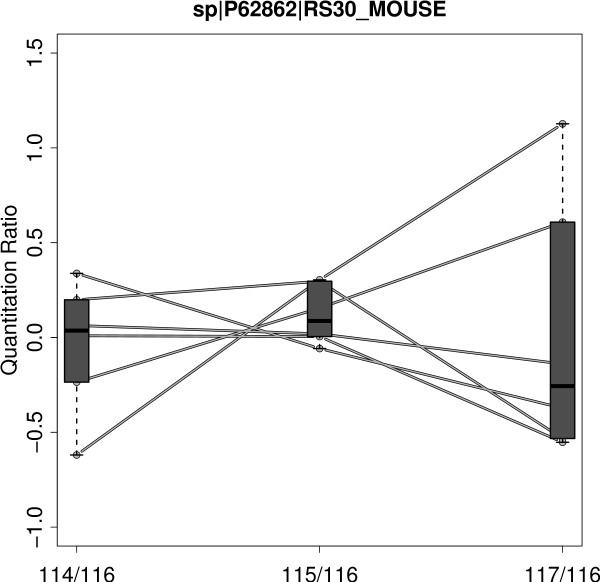
**Peptide Heterogeneity**. Exemplary chosen protein accession *40S ribosomal protein S30 (RS_30) *for demonstration of peptide heterogeneity. Every line represents a unique peptide profile (peptide-spectrum-match) identified as originating from the RS_30 protein. iTRAQ ratios are calculated using 116 channel (SJL mouse with standard diet) as reference. For every ratio a box plot giving the lower quartile, median and upper quartile is drawn. Especially for the 117/116 ratio (NZO mouse with high fat diet) the quantitation ratios are very heterogeneous ranging from -0.5 to +1 (corresponding to a 1.4 fold down-regulation or 2 fold up-regulation).

The continuing popularity of iTRAQ makes an evaluation of the technique in terms of accuracy and precision a valuable task [19]. Accuracy assesses the closeness to the real quantification value. Precision in this context refers to reproducibility of experiments. Since accuracy is difficult to evaluate, precision is the most frequently applied measure for experimental quality [20,21]. Gan et al. [22] tried to assess the precision of iTRAQ data by analyzing technical (different channels of the same MS run), experimental (same channel but different runs) and biological variations (different biological samples). They designed different iTRAQ experiments covering the different types of replications and they found technical variation to be small (11%) whereas experimental and biological variations where more than twice as high. For iTRAQ - like for the majority of MS based quantitation approaches - quantitation measurements are performed at the peptide level. Since often multiple peptides potentially with different modifications are measured for the same protein, the need for some kind of summarizing strategy is obvious. Different ideas regarding the calculation of protein quantitation from multiple peptides have been applied including mean or median calculation [23,24] and error weighted means [25]. Because of the fixed stoichiometric ratio, quantitation measurements for peptides uniquely assigned to the same protein should be strictly correlated [26]. But often this presumption is not fulfilled and the quantitation values exhibit a substantial heterogeneity. The heterogeneity is also observed for quantitation ratios and z-transformed values and is not due to different ionization or fragmentation efficiency. This is illustrated in Figure 1 presenting the quantitation ratios of unique peptides for an exemplary chosen protein: *40S ribosomal protein S30*. Especially the 117/116 ratio (rightmost bar in Figure 1) varies from 1.4 fold down-regulation to 2 fold up-regulation. An obvious reason for heterogeneous quantitation values are non-unique peptides shared by different proteins.

To correct for heterogeneity of peptides for the same protein, many approaches make use of outlier detection methods like Grubb's test [23] or Dixon's test [25] prior to averaging. However, for several reasons outlier filters are problematic: First, outlier filtering can be applied only to proteins with a certain minimum number of peptides, a presumption often not fulfilled in iTRAQ datasets [27]. Second, if heterogeneity is due to differentially regulated protein isoforms, the less frequent isoform is possibly regarded as an outlier and removed leading to loss of information. Third, if outlier detection is applied after protein inference, false positive peptides are removed that contributed to the protein identification score and hence the score is distorted a posteriori.

**Figure 2:**
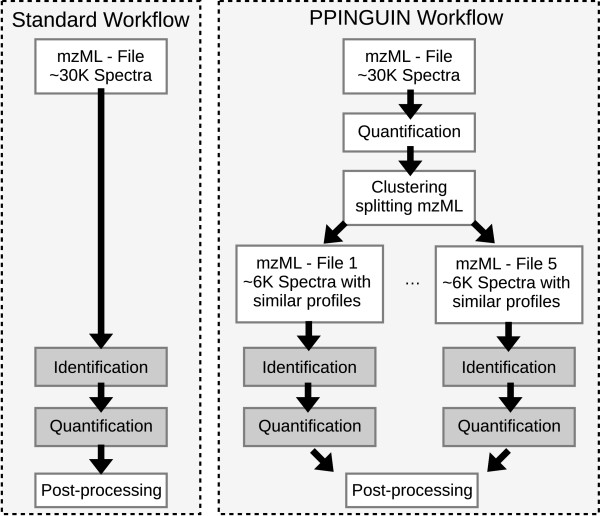
**Workflow**. Standard workflow of proteomics data evaluation (left hand side) compared to the PPINGUIN workflow presented in our manuscript (right hand side). Starting point for both workflows is the mzML [48] file containing the spectral peak data. In contrast to the standard workflow we employ clustering as a very early step prior to protein inference. This leads to splitting of spectra into different groups. Quantitation and identification is performed independently for each group. The result is a list of identified and quantified proteins ready for downstream analysis.

Here we present a statistical analysis workflow for iTRAQ data employing clustering prior to protein inference with the aim to reduce peptide heterogeneity (see Figure 2).

## Methods

### Dataset

Liver tissue samples from two different inbred mouse strains were investigated. The New Zealand Obese (NZO) mouse strain exhibits a polygenic obesity associated with hyperinsulinaemia and hyperglycaemia and presents additional features of a metabolic syndrome, including hypertension, and elevated levels of serum cholesterol and serum triglycerides [28]. In contrast, the Swiss Jim Lambert (SJL) mouse strain is lean and resistant to diet-induced obesity [29]. SJL mice carry a naturally occurring loss-of-function mutation in the TBC1D1 gene that confers leanness and protects from diet-induced obesity and diabetes [30]. In fact, deletion of TBC1D1 leads to elevated lipid oxidation in skeletal muscle that prevents weight gain in response to a high fat diet. Conversely, NZO mice are highly susceptible to weight gain when fed a high-fat diet, resulting in the development of morbid obesity, with fat depots exceeding 40% of total body weight [31].

After weaning at week 3, male NZO and SJL mice were raised on two different diets, a low fat diet (SD; 16% calories from fat) and a high fat diet (HF; 35% calories from fat). At week 12, mean body weight of SJL mice was 18.99 g (+/- 1.41 g) on SD and 20.1 g (+/- 1.42 g) on HF. In contrast, mean values for NZO mice were 38.81 g (+/- 1.85 g) on SD and 56.52 g (+/- 3.45 g) on HF, respectively. The mice were then sacrificed and liver tissue samples were analyzed.

Animals were kept in accordance with the NIH guidelines for the care and use of laboratory animals and all experiments were approved by the Ethics Committee of the State Ministry of Agriculture, Nutrition and Forestry, State of Brandenburg, Germany (23-2347-8-19-2008). Three to six mice per cage (macrolon type III) were housed at a temperature of 22°C and a 12 h light-dark cycle (lights on at 6 a.m.). Throughout the study the animals had free access to food and water.

Experimental design and iTRAQ labeling strategy are shown in Table 1. Three experimental replications were performed for each combination of genotype and diet. Experimental replications comprises a total of twelve different mouse individuals and four different iTRAQ channels (see Table 1). Due to this experimental design, the measured variance for each combination of genotype and diet is a superposition of technical (different iTRAQ channels) and biological error (different mouse individuals). This design was chosen to match real-life experiments where these errors are important.

**Table 1:** Experimental Design

	NZO_SD	NZO_HF	SJL_SD	SJL_HF
Exp 1	mouse:1 - channel:114	mouse:4 - channel:117	mouse:7 - channel:116	mouse:10 - channel:115
Exp 2	mouse:2 - channel:115	mouse:5 - channel:114	mouse:8 - channel:116	mouse:11 - channel:117
Exp 3	mouse:3 - channel:116	mouse:6 - channel:115	mouse:9 - channel:117	mouse:12 - channel:114

Experimental design and iTRAQ labeling (114 - 117) for three experimental replications (Exp 1, Exp 2 and Exp 3). For every distinct combination of genotype and diet 3 different mouse individuals are used.

The dataset (Mascot Generic Files - mgf) was uploaded to PRIDE [32] - Accession number: 20140.

### MASCOT

Peptide identification and quantitation were performed using MASCOT search engine (version 2.2.04 Matrix Science, London). Peptides identified with a MASCOT score < 50 and a significance threshold of *p *> 0.05 were neglected. Searches were performed using the following parameter set: Enzyme: Trypsin; maximum missed cleavages: 2; fixed modifications: Methylthio (C), iTRAQ4plex (N-term), iTRAQ4plex (K); quantitation method: iTRAQ 4 plex with weighted protein ratio and median normalization of ratios; variable modifications: Oxidation (M), iTRAQ4plex (Y); peptide mass tolerance: 10 ppm; fragment mass tolerance: 0.8 Da; mass values: monoisotopic; instrument type: ESI-FTICR; Isotope error mode: 0; minimum of 1 peptide per protein identification.

The database used was a SwissProt derived FGCZ in-house mouse database from 2009 containing 43636 mouse protein sequences (OS = Mus musculus) and 259 additional FGCZ specific entries. All proteins are present in normal/forward sequences and decoy/reverse sequences. Randomized decoy database (reversed sequences) was used for controlling false discovery rate (FDR) [33,34]. For calculation of FDR the list of proteins ordered by MASCOT *ProtScore *was cut when given FDR level was reached. Because we intend to achieve reliable quantitation results rather than provide a comprehensive protein list, the false discovery rate was chosen restrictively: FDR = 0.1%.

### X!Tandem and OpenMS

Peptide identification was performed using X!Tandem software (http://www.thegpm.org/tandem) [35] version 2009.04.01.1. X!Tandem search was performed using the following parameter set: cleavage site: '[RK]|P'; precursor-charge: 2; missed-cleavages: 2; fragment-mass-tolerance: 0.8 Da; precursor-mass-tolerance: 10 ppm; fixed-modifications: iTRAQ4plex (N-term), iTRAQ4plex (K), Methylthio (C); variable-modifications: Oxidation (M), iTRAQ4plex (Y); refinement of unanticipated cleavages.

Extraction of 4-plex iTRAQ quantitation data and isotope correction was performed using OpenMS (http://open-ms.sourceforge.net) [36,37] svn revision 6265. The same decoy database as for MASCOT analysis was used and again false discovery rate was chosen restrictively: FDR = 0.1%. For calculation of FDR the list of proteins ordered by X!Tandem protein identification score was cut when a given FDR level was reached.

### Peptide Profiling Guided Identification of Proteins - PPINGUIN

We define an iTRAQ quantitation profile of a spectrum as the ordered list of the raw quantitation values, in our case the raw intensities of the four iTRAQ channels 114 to 117. PPINGUIN seizes on the presumption that profiles of peptides derived from the same protein are highly correlated as they have a common source. As a first step and thus without regarding protein inference, iTRAQ quantitation profiles of the spectra are calculated by extracting the four quantitation values using OpenMS. In this prove of concept study, we want to show that clustering based on quantitation profiles representing different experimental conditions can help to correctly quantify proteins. In order to avoid distortions by missing values, we restrict the analysis to spectra with complete quantitation profiles and remove spectra with incomplete profiles. The recommended isotope correction is performed according to manufacturer's specifications (Applied Biosystems, Foster City, CA) using OpenMS. Isotope correction aims at correcting for trace levels of isotopic impurities and is done by solving a system of equations. In addition a complementary normalization of the four quantitation values is performed as described below.

Logarithmic quantitation profiles of the spectra are clustered in a coarse-grained manner using k-means algorithm [38] based on Euclidean distance and randomly selected starting points. We use k-means clustering (k = 5) as it is computationally fast and well suited to demonstrate the benefit of the pre-selection. The group size parameter k = 5 was chosen according to two internal cluster validation measures (see Section 'Number of Clusters'). To analyze stability of the clustering, it was performed for 1000 replications each with different randomly chosen starting points. From 1000 iterations 999 resulted in the same or a very similar partitioning of the quantitation profiles of the spectra.

Clustering intends to create groups of peptides with similar biological profiles (e.g. up-regulation for a certain combination of genotype and diet). As subsequent analysis is focused on relative iTRAQ ratios instead of absolute quantitation values and Euclidean distance is not scale independent, the profiles are centered prior to clustering (mean is set to zero). Euclidean distance used as distance measure clustering is not scale independent. In order to preserve differences between relative iTRAQ ratios no additional scaling was performed (standard deviation is preserved). This procedure equals to a clustering using Euclidean distances on centered logarithmic quantitation profiles. With this procedure an explicit choice of a reference channel is not necessary. Every spectrum is assigned to exactly one group and for every group the corresponding spectra show similar quantitation profiles. Quantitation and identification is now performed independently for every group with identical settings to X!Tandem and OpenMS approach. Similar to the X!Tandem/OpenMS approach, FDR was calculated by cutting the list of proteins ordered by X!Tandem protein identification score if a given FDR level was reached. The FDR is calibrated for each group individually and in effect, X!Tandem threshold for protein identification differs in each group. Finally, *log*_2 _ratio profiles are calculated using SJL genotype with standard diet (SD) as reference. Following the definition of iTRAQ quantitation profiles, ratio profiles are defined as the list of 3 possible iTRAQ ratios (e.g. for Exp 1: 114/116, 115/116 and 117/116 - see Figure 1).

All calculations (normalization and clustering) were performed using R statistical programming language (R version 2.7.0 - 2008-04-22). The R-script of our implementation of PPINGUIN is provided as Additional File 1. Protein inference and extraction of quantitation values was performed using X!Tandem and OpenMS as described previously.

### Normalizing iTRAQ quantitations

Additional normalization of the 4 quantitation values is required to correct for technical bias [19]. Karp et al. [27] observed a heterogeneity of variance for iTRAQ ratios where the width of the distribution is significantly larger at low intensities. They proposed a variance stabilizing normalization based on VSN software [39]. We compared three different normalization strategies: VSN, multi lowess algorithm - a multi dimensional extension of lowess normalization strategy [40] and median correction. In our dataset we see heterogeneity of variance for unnormalized data as well as for median corrected data. The other two normalization approaches lead to an almost constant variance (see Additional File 2 for more details). We selected multi-lowess as our preferred normalization strategy.

### Number of Clusters

The number of clusters is an important parameter for clustering. The preferable number of clusters was determined using two different internal measures: gap statistic [41,42] and Xie-Beni index [43]. Both measures were calculated for 25 repetitions of runs. The preferable number of clusters was determined to be in the range between 3 and 7. Therefore, we selected 5 as a reasonable number of clusters.

### Calculation of CV values for Peptide Homogeneity

Let *y*_*j,r *_be the relative quantitation ratio for a peptide j and ratio *r *∈ *R *= {NZO_SD/SJL_SD, NZO_HFD/SJL_SD and SJL_HFD/SJL_SD }. To assess peptide homogeneity, we calculate the coefficient of variation of a protein *p *by using all unique peptides for proteins:

$$CV_{p}=\frac{1}{3*n_{p}}\underset{j\in p}{\sum }\underset{r\in R}{\sum }\frac{\sigma_{j,r}}{\mu_{j,r}}$$

where *n*_*p *_is the number of unique peptides for protein p and *σ*_*j,r *_and *μ*_*j,r *_are the standard deviation and mean of relative quantitation ratios *y*_*i,r *_of all peptides uniquely assigned to protein p. The final coefficient of variation is calculated by averaging *CV*_*p *_for all proteins.

### Calculation of CV values for Experimental Reproducibility

Let *y*_*e,i,r *_be the relative quantitation ratio for experiment *e *∈ {Exp1, Exp2, Exp3}, protein *i *∈ *I *= 1..*n *and ratio *r *∈ *R *= { NZO_SD/SJL_SD, NZO_HFD/SJL_SD and SJL_HFD/SJL_SD }. In order to assess experimental reproducibility of *r *we calculate the average CV of all proteins occurring in all three experiments:

$$CV_{r}=\frac{1}{n}\cdot \underset{i\in I}{\sum }\left(\frac{\sigma_{i,r}}{\mu_{i,r}}\right)$$

where *σ*_*i,r *_and *μ*_*i,r *_are the standard deviation and mean of relative quantitation ratios *y*_*i,r *_for protein *i *and ratio *r *for all three experiments:

$$\begin{array}{c} \mu_{i,r}=\frac{1}{3}\sum_{e\in E}y_{e,i,r}\\ \sigma_{i,r}=\sqrt{\frac{1}{2}{\sum_{e\in E}\left(y_{e,i,r}-\mu_{i,r}\right)}^{2}} \end{array}$$

This value is reported in Table 2 together with mean standard deviation of *log*_2 _ratios:

**Table 2:** Experimental Reproducibility

	Ratio	MASCOT	X!Tandem/OpenMS	PPINGUIN
	NZO_SD/SJL_SD	0.13	0.12	0.10
CV	NZO_HFD/SJL_SD	0.17	0.16	0.14
	SJL_HFD/SJL_SD	0.18	0.17	0.15

	NZO_SD/SJL_SD	0.19	0.17	0.14
StDev *log*_2_	NZO_HFD/SJL_SD	0.25	0.22	0.20
	SJL_HFD/SJL_SD	0.24	0.24	0.21

Experimental reproducibility using the analysis methods investigated (columns). For the 3 experimental ratios (NZO_SD/SJL_SD, NZO_HFD/SJL_SD and SJL_HFD/SJL_SD) the mean coefficient of variation (CV) and the mean standard deviation for *log*_2 _quantitation ratios (see Methods) of all proteins are stated.

$$StDev_{r}=\frac{1}{n}\cdot \underset{i\in I}{\sum }\left({\hat{\sigma }}_{i,r}\right)$$

where ${\hat{\sigma }}_{i,r}$ is the standard deviation of *log*_2 _ratios:

$$\begin{array}{l} \;\;{\hat{\mu }}_{i,r}=\frac{1}{3}\sum_{e\in E}log_{2}(y_{e,i,r})\\ {\hat{\sigma }}_{i,r}=\sqrt{\frac{1}{2}\sum_{e\in E}{\left(log_{2}(y_{e,i,r})-{\hat{\mu }}_{i,r}\right)}^{2}} \end{array}$$

## Results

We present a novel workflow termed Peptide Profiling Guided Identification of Proteins (PPINGUIN). PPINGUIN proceeds by first clustering spectra based on their quantitation values and than inferencing proteins for each cluster independently (see Methods). The results of our approach are compared with standard evaluation approaches using MASCOT and X!Tandem/OpenMS software (see Methods).

### Proteins identified

**Figure 3:**
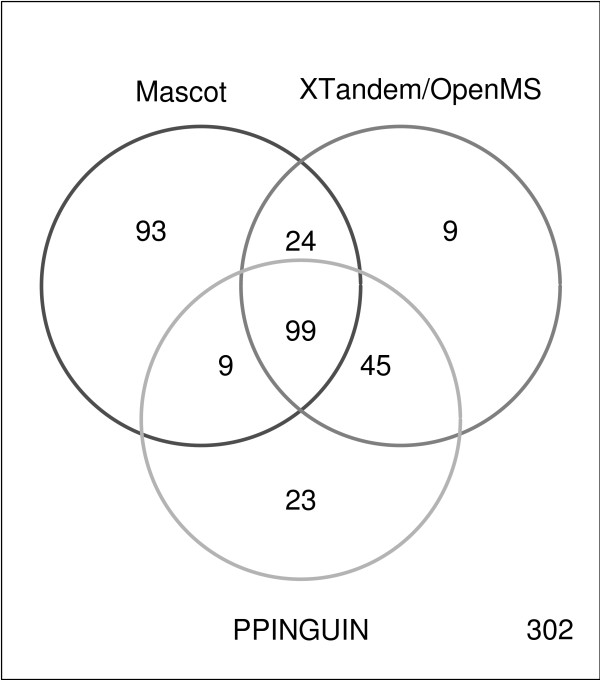
**Venn Diagram**. Venn diagram visualizing the number of significantly identified protein accessions using the three different approaches: Mascot, XTandem/OpenMS and PPINGUIN. We refer to protein accessions identified in all three experimental replications of the diabetes dataset (see Methods).

The numbers of protein accessions identified with the same FDR (see Methods) differ for each method: 225 for MASCOT, 177 for X!Tandem and OpenMS based approach and 176 for PPINGUIN. Ambiguous protein groups (e.g. H2B1B, H2B1C, H2B1F,...) identified with exclusively non-unique peptides, were not counted here. Therefore, the actual number of proteins and the overlaps of the three methods may be underestimated. Most of the representative accessions received from PPINGUIN analysis were also detected using X!Tandem (83%). Both methods have their set of unique proteins: 32 for PPINGUIN and 33 for X!Tandem. The overlap between MASCOT and the other two approaches is good: 70% of the X!Tandem IDs and 62% of PPINGUIN IDs were found with MASCOT (see Venn diagram in Figure 3). Explanations for these differences are provided in the discussion below.

The quality of the three methods is assessed in terms of three different criteria: (i) homogeneity of peptide profiles (ii) reproducibility in independent experiments and (iii) accordance with prior knowledge.

### Homogeneity of peptide profiles

**Figure 4:**
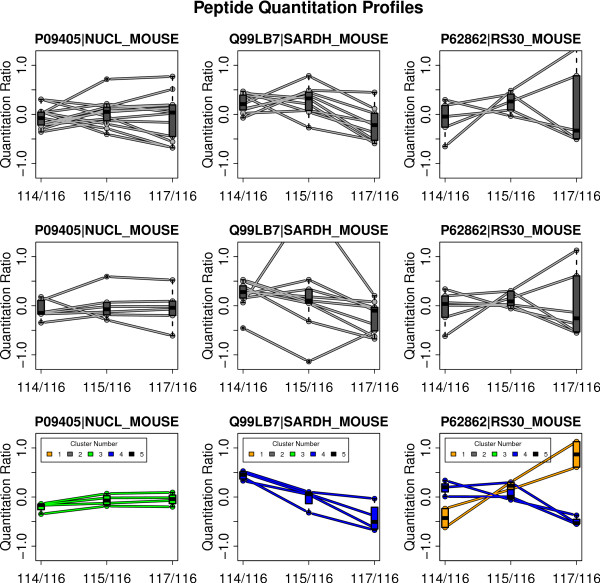
**Sample peptide profiles**. Visualization of peptide quantitation profiles of the three different approaches employed (rows) demonstrated for 3 exemplary chosen proteins (columns). The three rows correspond to the applied method: first row = MASCOT, second row = X!Tandem and OpenMS, last row = PPINGUIN. Each individual plot shows ratio profiles of peptides uniquely assigned to the corresponding protein. For every ratio a box plot giving the lower quartile, median and upper quartile is drawn.

**Figure 5:**
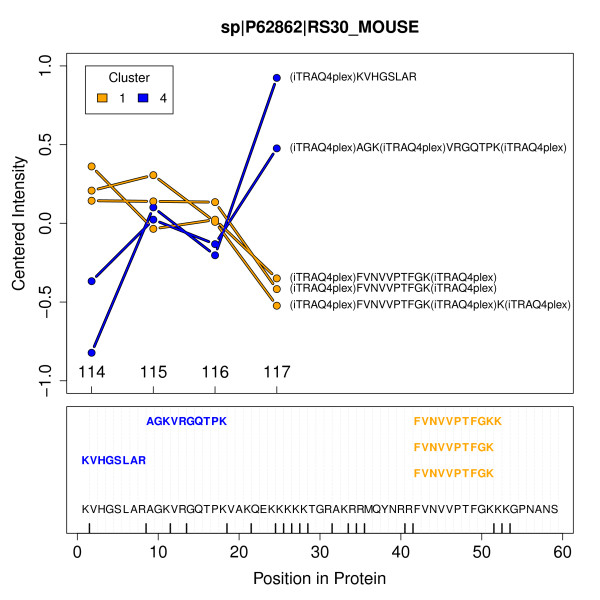
**Ribosomal Proteins**. Upper part: Quantitation profiles of the unique peptides assigned to the ribosomal protein RS30 detected in the first experiment. Labels are representing the samples: 114 - NZO_SD; 115 - SJL_HF; 116 - SJL_SD and 117 - NZO_HF. Colors orange and blue correspond to clusters 1 and 4 the peptides were identified in. Lower part: Protein sequence with positions of mapped peptides. Vertical bars displayed on the x-axis indicate predicted trypsin cleavage sites. The non-tryptic peptide was found with X!Tandem option 'refine unanticipated cleavages'.

As described above, a protein represented by multiple unique peptides should result in strictly correlated quantitation ratios for the peptides. But often heterogeneous ratio profiles are observed using MASCOT as well as X!Tandem, naturally leading to difficulties in quantitative interpretation. This situation is illustrated in the first and second row of Figure 4 for three example proteins. An obvious reason for heterogeneous quantitation values are non-unique peptides shared by different proteins. For avoiding this fact non-unique peptides are left out for all plots and statistical assessments. Using our approach, peptide profiles are more homogeneous supporting a consistent quantitative interpretation (see bottom row of Figure 4). A distinctive feature of PPINGUIN is demonstrated by the ribosomal protein RS_30: inconsistent quantitation profiles are resolved by splitting up in two groups each with homogeneous profiles. This effect is illustrated in more detail in Figure 5 (and as addition examples in Additional File 3). The protein is identified in two different clusters (1 and 4) with different peptide profiles. The peptides in cluster 1 show low relative concentration for NZO_SD (114) and high relative concentration for NZO_HF (117) while peptides in cluster 4 show the opposite behavior. The peptides belonging to each cluster are located in different sites of the protein. As discussed below, this finding is a hint towards two variants of the RS_30 protein.

For quantitative assessment of overall peptide profile homogeneity we have performed a comprehensive statistical evaluation. We calculated CV values for each of the three approaches (see method section). We observed a CV of peptides assigned to a protein of 20% for MASCOT and 26% for X!Tandem while PPINGUIN has a smaller CV of 14%.

Reduced variance in PPINGUIN is an expected effect since the peptides within each cluster are rather similar by construction. For an independent assessment of our method we now proceed to investigate experimental reproducibility (precision) and accordance with prior knowledge.

### Precision - Experimental Reproducibility

In order to test reproducibility we performed three independent experimental replications and three independent evaluations (see Dataset section in Methods). We investigated two different mouse genotypes and two diets resulting in 4 distinct combinations. The 4 combinations define 3 ratios using SJL genotype with standard diet (SD) as reference. Quantitation ratios for a protein are calculated by averaging the log ratios of the corresponding peptides. To facilitate comparability we restrict the analysis to the set of 99 proteins identified in all three experimental replications.

We calculated mean coefficient of variation and mean standard deviation of log quantitation ratios (CV and StDev - see Methods section) of all proteins (see Table 2).

The analysis was performed separately for each of the 3 experimental ratios: NZO_SD/SJL_SD, NZO_HFD/SJL_SD and SJL_HFD/SJL_SD.

Experimental variation of the MASCOT based evaluation is characterized by CV values ranging from 0.13 to 0.18 (see first column in Table 2). X!Tandem/OpenMS results in CV values ranging from 0.12 to 0.17 (second column in Table 2). Experimental variation is reduced using PPINGUIN with CV values ranging from 0.10 to 0.15 (third column in Table 2).

Different from the improved homogeneity in the previous section, the lower error of PPINGUIN is not a trivial effect since the complete analysis workflow is performed for each experiment independently. These results demonstrate that applying the proposed method for data evaluation leads to more stable quantitation values.

### Accordance with prior knowledge

The identification of differentially expressed proteins is a major goal of quantitative proteomics. We now compare the set of differentially expressed biomarker candidates obtained with the three different methods. To assess the results of the differential analysis, we use a set of 'gold standard' genes identified in the context of type-2 diabetes [44]. This meta-analysis reports top gene candidates for mixture of genotypic and dietary effects. To achieve comparability with the meta-analysis, differential analysis is performed comparing NZO mice with high fat diet and SJL mouse with standard diet (see Dataset section).

Top lists of differentially expressed proteins are created by selecting proteins with mean absolute *log*_2 _fold changes above arbitrarily chosen threshold of 0.5 (1.4 fold change). Due to the low number of replicates we use the fold instead of the p-value as criterion to judge differential expression.

Evaluation based on MASCOT identifies a total of 14 differentially regulated proteins of which 29% (4) are found in the reference. Using X!Tandem and OpenMS we identified only 8 differential proteins of which 37% (3) are found if the reference set. PPINGUIN results in 14 differentially expressed proteins, of which 50% (7) are part of the reference set. Table 3 presents the statistics of the differentially regulated proteins identified using PPINGUIN (proteins of the reference set are marked with asterisks). Of the three methods, PPINGUIN shows the highest agreement with the reference list. This remains true for alterations of the threshold value (e.g. 0.3 or 0.7).

**Table 3:** Accordance with prior knowledge

Protein ID	Description	***log***_**2 **_**Fold**	P-Value	#Peptides	X!Tandem Score
Q9Z204	heterogeneous nuclear ribonucleoprotein C	1.21	0.158	2	2.8
O35490	betaine-homocysteine methyltransferase	-0.979	0.00148	24	59.6
P33267	cytochrome P450, family 2, subfamily f*	-0.857	0.131	3	10.1
P97872	flavin containing monooxygenase 5	-0.799	0.0425	3	10.6
Q91V92	ATP citrate lyase*	0.72	0.231	5	9.4
Q9Z2V4	phosphoenolpyruvate carboxykinase 1*	-0.706	0.0782	2	6.8
Q8VCH0	acetyl-Coenzyme A acyltransferase 1B	0.693	0.0318	2	6.1
P10649	glutathione S-transferase, mu 1*	-0.689	0.105	5	8.3
P01942	hemoglobin alpha, adult chain 1	0.678	0.359	16	16.2
P70694	aldo-keto reductase family 1*	-0.634	0.0245	6	17.7
Q9CPY7	leucine aminopeptidase 3	-0.629	0.0926	4	17.5
Q8R0Y6	aldehyde dehydrogenase 1 family, member L1	-0.566	0.0747	5	15.6
P12710	fatty acid binding protein 1*	0.524	0.221	17	50.8
P53657	pyruvate kinase liver and red blood cell*	0.51	0.278	8	35.3

Top list of differentially regulated proteins identified using PPINGUIN. Proteins marked with an asterisk (*) have previously been associated with diabesity [44]. P-values are calculated using one-sample t-test (null hypothesis: *log*_2_(NZO_HFD/SJL_SD) = 0). P-values are not used as a criterion for differential expression and are not corrected for multiple testing. With an increasing number of replicates in future studies significance of the p-values may be improved.

## Discussion

Typically, data mining techniques are applied after protein inference and quantitation. In contrast to the standard workflow, our approach employs clustering prior to protein inference as a very early step in data processing (see workflow comparison in Figure 2). Recently different approaches have been proposed to improve protein identification using peak intensities [26,45]. In contrast to these works, our major goal is to improve quantitation itself based on a set of proven and tested identification tools.

A key feature of our approach is shown in Figure 5: the separation of unique peptides for a protein in multiple clusters. Non-unique peptides shared by different proteins are not considered. The peptides in each cluster exhibit distinct quantitation profiles which are most likely corresponding to protein isoforms. Typical reasons for isoforms are protein modification, splice variants or degradation effects.

For further investigation of protein modifications, we first identified most frequent modifications in our dataset. For each of the 800 modifications listed in Unimod [46] we re-performed protein inference searching for single variable modifications. The most frequent modification found was oxidation of methionine which increased the number of found peptide-spectrum-matches by almost 10%. Oxidation of methionine, whose impact on iTRAQ has been reported previously [47], can be caused by an enzymatic reaction but can also be due to sample preparation in the presence of reactive oxygen species. Other frequent modifications were 'Oxidation (D)', 'Oxidation (N)', 'Deamidated (Q)'. Subsequently we re-performed the analysis allowing for these 4 variable modifications simultaneously. However, in this second identification step we did not find further evidence for protein modification regarding the RS_30 protein isoforms.

Investigating splice variants as a possible explanation, we found that RS_30 protein is transcribed from exon 4 and 5 of the FAU (Ensembl-ID: ENSMUSG00000038274) gene. The peptides from different clusters are located in different regions of the protein which also correspond to the different exons of the FAU gene, but there was no indication for differential splicing in the database. However, the FAU gene may have two variants: the RS_30 protein with 59 amino acids and the completely transcribed protein with 133 amino acids. PPINGUIN finds two variants of the RS 30 gene. The two isoforms found by PPINGUIN may correspond to the two potential variants, which of cause would require further experimental investigations. But if PPINGUIN can detect potential novel splice variants it may help to improve protein or even nucleotide databases.

PPINGUIN is not designed to exploit known protein variants, but it may indirectly re-detect also known variants. Incorporating the knowledge of known protein variants during protein inference, should further improve protein quantitation.

The set of identified proteins is altered comparing PPINGUIN and X!Tandem. Assignment of peptides to different groups and subsequent protein identification for each group individually, is expected to lower identification significance and thus to reduce the number of proteins. Indeed, a random grouping identifies only 138 (*±*10) proteins in all experiments. However, biologically motivated clustering used by PPINGUIN, leads to a total of 176 proteins, 32 of which are found only by PPINGUIN and not by X!Tandem. This is due to two combined effects: First, exploiting quantitation profile information, our clustering leads to a relative enrichment of peptides belonging to the same protein in a cluster and second, by splitting spectra into groups, clustering decreases the total number of spectra in each identification process. The reduced number of spectra per cluster alters the identification threshold used for calibration of the false discovery rate and in effect new proteins are identified. The largest set of uniquely identified proteins was found for MASCOT. Most of these 93 unique MASCOT proteins are also found using X!Tandem but they remain below the significance threshold. This is mostly due to differences in the assessment of short peptides since MASCOT appears to include many small peptides for identification that are excluded by X!Tandem. The set of quantified protein accessions received by PPINGUIN is characterized by an increased experimental reproducibility compared to the other methods. This implies that using PPINGUIN for evaluation, one experimental outcome is a more reliable predictor for the outcome of a similar experiment. Finally, the comparison with prior knowledge showed a surprisingly high agreement of our top proteins with a reference set, which we deem representative for diabetes and obesity. This hints for the practical benefit of our method.

## Conclusion

We proposed a novel method for evaluation of iTRAQ data motivated by the observation that relative concentrations of peptides derived from the same protein often show unexpectedly heterogeneous correlation patterns. Exploiting correlations of quantitation ratios achieves more consistent quantitation ratios than the standard approaches. This is demonstrated by an increased reproducibility of independent experiments. Besides leading to a more reliable quantitation, the method can reveal new isoform candidates.

We see our work as a promising step towards quantitation guided identification. In general, we recommend to use our method in case accurate quantitation is a major objective of research. Regarding the increasing importance of quantitative proteomics we think that this method will be useful in practical applications like model fitting or functional enrichment analysis.

We expect that our approach will be still more valuable with an increasing number of parallel quantified samples (e.g. 8-plex iTRAQ) since the importance of the clustering increases. The proposed approach can also be very useful for other quantitative proteomics technologies like e.g. SILAC. A next step will be to extend the algorithms to include spectra with incomplete iTRAQ quantitations. Future versions of PPINGUIN will aim at further refinement of protein quantitation by incorporating the rapidly growing public knowledge on splice variants and protein isoforms.

## Authors' contributions

CB developed and implemented the described methods and drafted the manuscript. TD, AC and HA were responsible for the generation of the mouse samples. DR and RS acquired the iTRAQ profile data. All authors read and approved the final manuscript.

## Supplementary Material

Additional file 1 **R-script of PPINGUIN**. PPINGUIN.R: R-script with our implementation of PPINGUIN. The script requires OpenMS and X!Tandem to be installed. Click here for file

Additional file 2 **Normalization - Results**. More detailed description of the normalization strategy applied in this work. The effects of the normalization algorithms on channel bias and homoscedasticity are demonstrated. Click here for file

Additional file 3 **Examples for Potential Isoforms**. Additional zip archive containing pdf images for 6 further examples with potential protein isoforms. Click here for file
