# Supplementary material for: PPINGUIN: Peptide Profiling Guided Identification of Proteins improves quantitation of iTRAQ ratios
Source: BMC Bioinformatics. 2012 Feb 16;13:34. doi: 10.1186/1471-2105-13-34 (PMC3368728; doi:10.1186/1471-2105-13-34)
Supplement: Additional file 2 — Normalization - Results. More detailed description of the normalization strategy applied in this work. The effects of the normalization algorithms on channel bias and homoscedasticity are demonstrated. [file 1471-2105-13-34-S2.PDF]

Normalization - Results

Typically raw iTRAQ data is biased by channel effects leading to differences in median quantitation values for each channel. This bias is visualized in the box plot of raw iTRAQ quantitation data exemplary for the first experiment (upper left part of Figure 1). This bias is comparable to fluorescent dye bias for DIGE or multi-color microarray data. Without correction, these differences lead to a systematic bias in quantitation ratios. The medians of the iTRAQ channels span a range of 0.85 which would correspond to 1.8-fold differential expression if not corrected. A normalization strategy aims to remove this systematic effect. Three different normalization algorithms were applied: vsn [1], multi-lowess [2] and median correction. All three methods successfully removed the channel bias and the differences in median quantitation value vanished (see Figure 1).

Another purpose of normalization is to assure homoscedasticity: homogeneity of variance. Homoscedasticity is a prerequisite for many statistical tests such as t-test or ANOVA. Figure 2 shows a standard error plot prior to and after application of the different normalization approaches. Prior to normalization the mean error of the 4 iTRAQ channels is 42% and the variance is lower for small intensities. After normalization the mean error of the 4 channels is only half as high: 22% for all normalization strategies applied. Median correction does not result in homoscedastic data since the error is higher for smaller quantitation ratios. Lowess and VSN normalization result in stable variances where the error is the same for small and high intensities. This observation is in accordance with the work of Karp et al. [3]. For our dataset, the other two normalization approaches lead to an almost constant variance and hence to homoscedasticity. Both, multi-lowess and VSN are very similar in terms of error and homogeneity of variance. Therefore we decided to use multi-lowess normalized data for further analysis.

References

1. Huber W, von Heydebreck A, Sultmann H, Poustka A, Vingron M: **Variance stabilization applied to microarray data calibration and to the quantification of differential expression**. *Bioinformatics* 2002, **18 Suppl 1**:96–104.

2. Quackenbush J: **Microarray data normalization and transformation**. *Nat. Genet.* 2002, **32 Suppl**:496–501.

3. Karp NA, Huber W, Sadowski PG, Charles PD, Hester SV, Lilley KS: **Addressing accuracy and precision issues in iTRAQ quantitation**. *Mol Cell Proteomics* 2010.

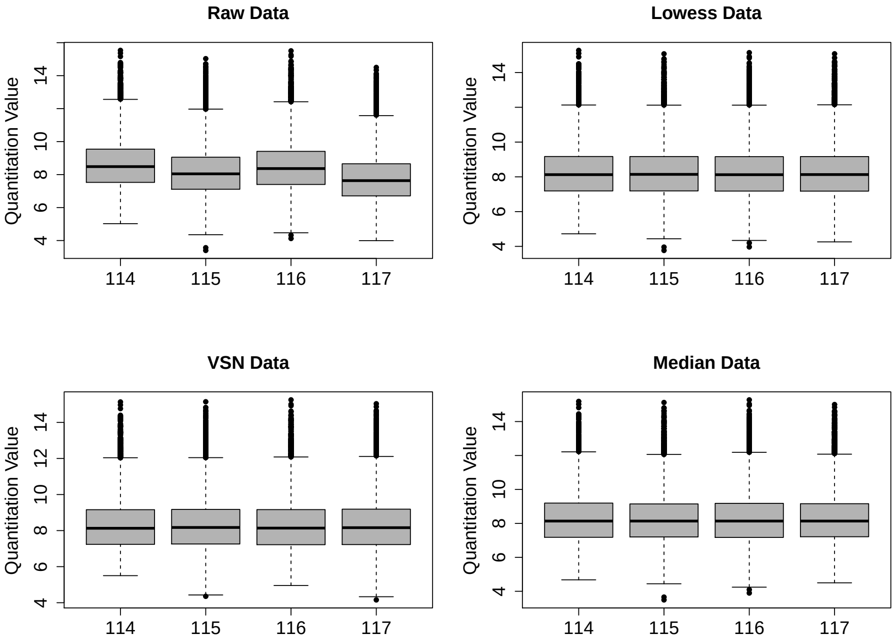

Supplemental Figure 1: Box plot of iTRAQ quantitation data before (upper left) and after application of three different normalization strategies: vsn (lower left), multi-lowess (upper right) and median correction (lower right). Prior to normalization there is a difference in median quantitation values that is removed by the normalization.

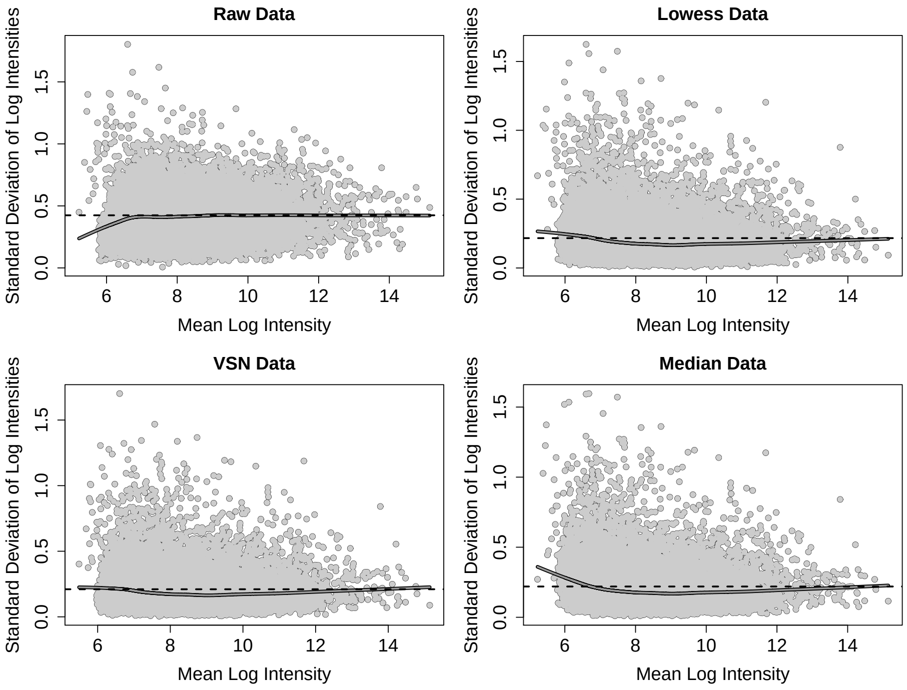

Supplemental Figure 2: Standard error plot of raw data (upper left) and after application of three different normalization strategies: vsn (lower left), multi-lowess (upper right) and median correction (lower right). Bold line represents a lowess fit and black dashed line reflect median standard deviation.
