# Supplementary figures and images for: PPINGUIN: Peptide Profiling Guided Identification of Proteins improves quantitation of iTRAQ ratios
Source: BMC Bioinformatics. 2012 Feb 16;13:34. doi: 10.1186/1471-2105-13-34 (PMC3368728; doi:10.1186/1471-2105-13-34)

**sp|P62806|H4\_MOUSE**

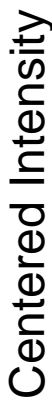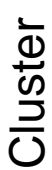

Supplement: Additional file 3 — Examples for Potential Isoforms. Additional zip archive containing pdf images for 6 further examples with potential protein isoforms. [file 1471-2105-13-34-S3.ZIP › ClusterPlotsP62806.pdf]

sp|Q64374|RGN\_MOUSE

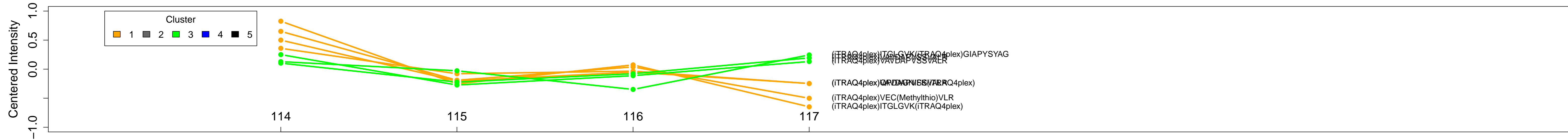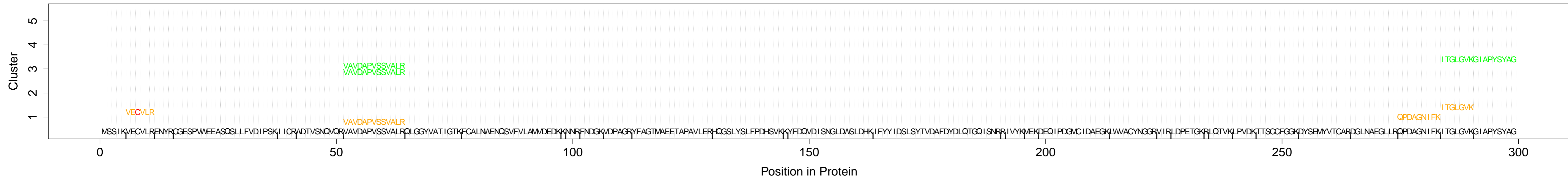

Supplement: Additional file 3 — Examples for Potential Isoforms. Additional zip archive containing pdf images for 6 further examples with potential protein isoforms. [file 1471-2105-13-34-S3.ZIP › ClusterPlotsQ64374.pdf]

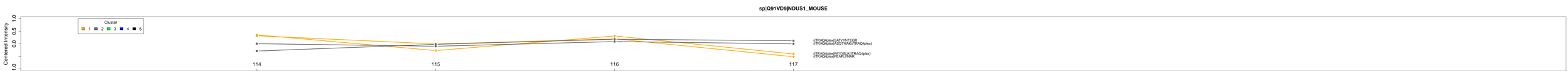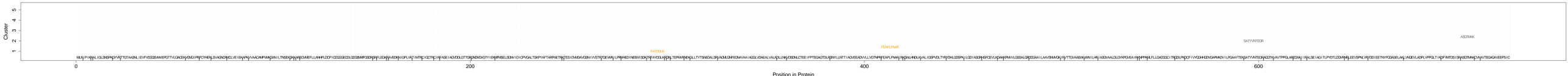

Supplement: Additional file 3 — Examples for Potential Isoforms. Additional zip archive containing pdf images for 6 further examples with potential protein isoforms. [file 1471-2105-13-34-S3.ZIP › ClusterPlotsQ91VD9.pdf]

sp|Q9CQS8|SC61B\_MOUSE

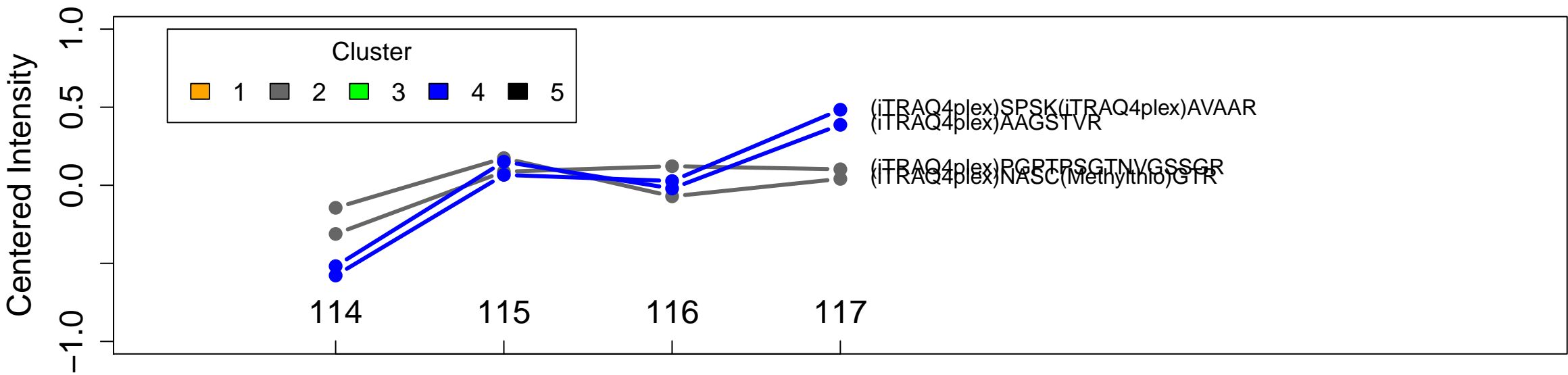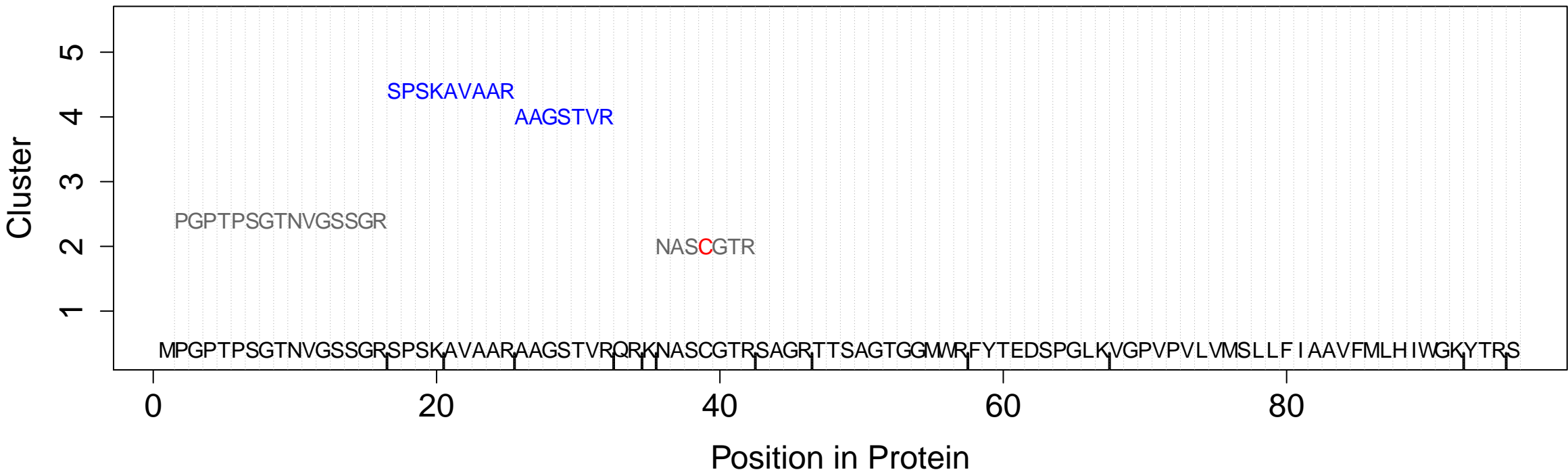

Supplement: Additional file 3 — Examples for Potential Isoforms. Additional zip archive containing pdf images for 6 further examples with potential protein isoforms. [file 1471-2105-13-34-S3.ZIP › ClusterPlotsQ9CQS8.pdf]
