# Supplementary material for: PPINGUIN: Peptide Profiling Guided Identification of Proteins improves quantitation of iTRAQ ratios
Source: BMC Bioinformatics. 2012 Feb 16;13:34. doi: 10.1186/1471-2105-13-34 (PMC3368728; doi:10.1186/1471-2105-13-34)

sp|Q6NZJ6−1|IF4G1_MOUSE

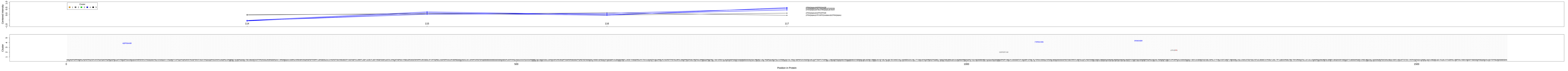

Supplement: Additional file 3 — Examples for Potential Isoforms. Additional zip archive containing pdf images for 6 further examples with potential protein isoforms. [file ClusterPlotsQ6NZJ6-1.pdf › ClusterPlotsQ6NZJ6-1.pdf]
