# Supplementary material for: PPINGUIN: Peptide Profiling Guided Identification of Proteins improves quantitation of iTRAQ ratios
Source: BMC Bioinformatics. 2012 Feb 16;13:34. doi: 10.1186/1471-2105-13-34 (PMC3368728; doi:10.1186/1471-2105-13-34)
Supplement: Additional file 3 — Examples for Potential Isoforms. Additional zip archive containing pdf images for 6 further examples with potential protein isoforms. [file ClusterPlotsQ8C196.pdf › ClusterPlotsQ8C196.pdf]

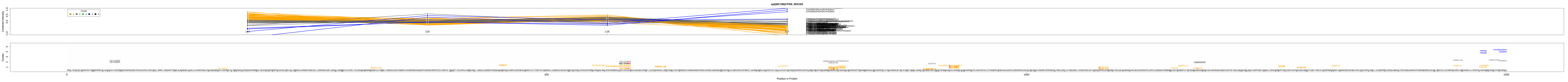

sp|Q8C196|CPSM_MOUSE
Centered Intensity
Cluster
Position in Protein
